# Dengue associated choroiditis: a rare entity

**DOI:** 10.1186/s12348-017-0132-5

**Published:** 2017-05-30

**Authors:** Harshali Manish Yadav, Parthopratim Dutta Majumder, Jyotirmay Biswas

**Affiliations:** 1 Department of Ophthalmology, Kerala Institute of Medical Sciences, Anayara P.O, Trivandrum, Kerala 695029 India; 2 Medical and Vision Research Foundations, Sankara Nethralaya, 18, College Road, Chennai, Tamil Nadu 600 006 India; 3 Uveitis & Ocular Pathology Department, Sankara Nethralaya, 41, College Road, Nungambakkam, Chennai, Tamil Nadu 600 006 India

**Keywords:** Dengue, Choroiditis

## Abstract

**Background:**

We report a case of choroiditis during dengue fever.

**Results:**

A 35-year-old female presented with blurring of vision during dengue fever. Her fundus examination revealed yellow deep choroidal lesions at right macula, multiple small, yellowish subretinal macular dots along papillomacular bundle, and hyperemic disc. There was a small retinal hemorrhage at temporal margin of disc. The left eye had similar small discrete lesion superonasal and inferotemporal to macula.

**Conclusions:**

We report a hitherto undescribed case of choroiditis in a dengue patient.

Dengue fever is a mosquito-borne *Flavivirus* infection in humans. The disease is endemic in the tropics, warm temperate regions of the world such as such as India, Southeast Asia, America, and the Western Pacific [1]. Dengue eye disease has plethora of presentations and includes subconjunctival hemorrhage, anterior uveitis, intermediate uveitis, maculopathy, chorioretinitis, neuroretinitis, and panuveitis [1]. We describe here an interesting case of a young lady who presented with choroidal involvement during dengue fever.

## Case report

**Fig. 1:**
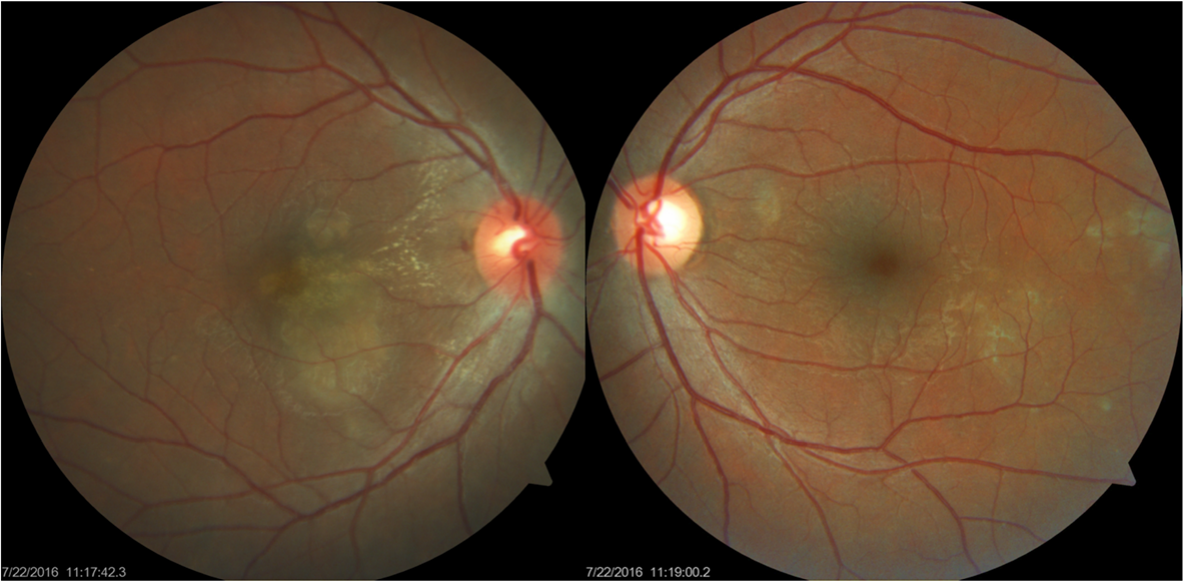
Fundus photographs of both the eyes at presentation. In the *right eye*, mild disc hyperaemia as compared to the *left eye* is noted. There is presence of punctuate retinal hemorrhage at temporal disc margin with hard exudates along papillomacular bundle. Multifocal yellow deep choroidal lesions are noted nasal to fovea. The *left eye* fundus picture shows normal disc with hard exudates superonasal and inferotemporal to macula

**Fig. 2:**
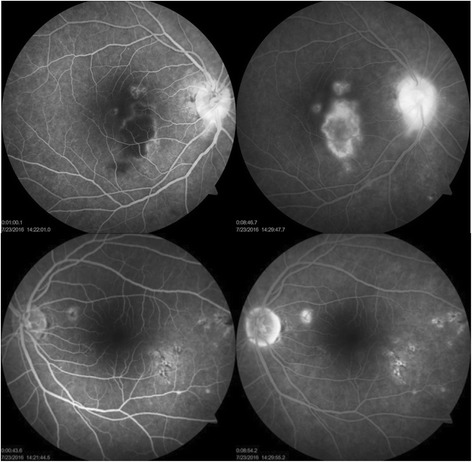
FFA in the right eye showed presence of three hypofluorescent lesions in posterior pole in early frames with progressively increasing hyper fluorescence in late frames due to leakage. Significant disc leakage is also noted. In the *left eye*, similar smaller hypofluorescent lesion is noted along papillomacular bundle with early central hypoflorescence and later hyperfluorescence. Area of stippled fluorescence was noted inferotemporal to macula with staining in late frames. Minimal disc leakage was also noted in the *left eye*

**Fig. 3:**
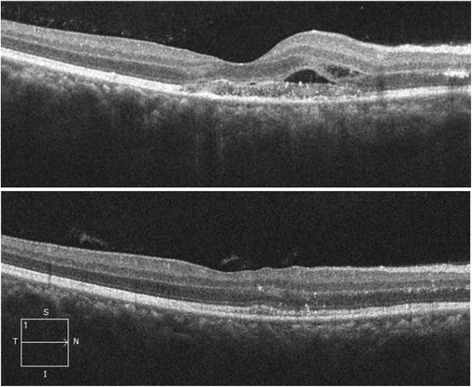
Pre and post-treatment OCT picture of the *right eye*. Pre-treatment OCT showing disruption and thickening of IS-OS junction with subretinal fluid and intraretinal edema

**Fig. 4:**
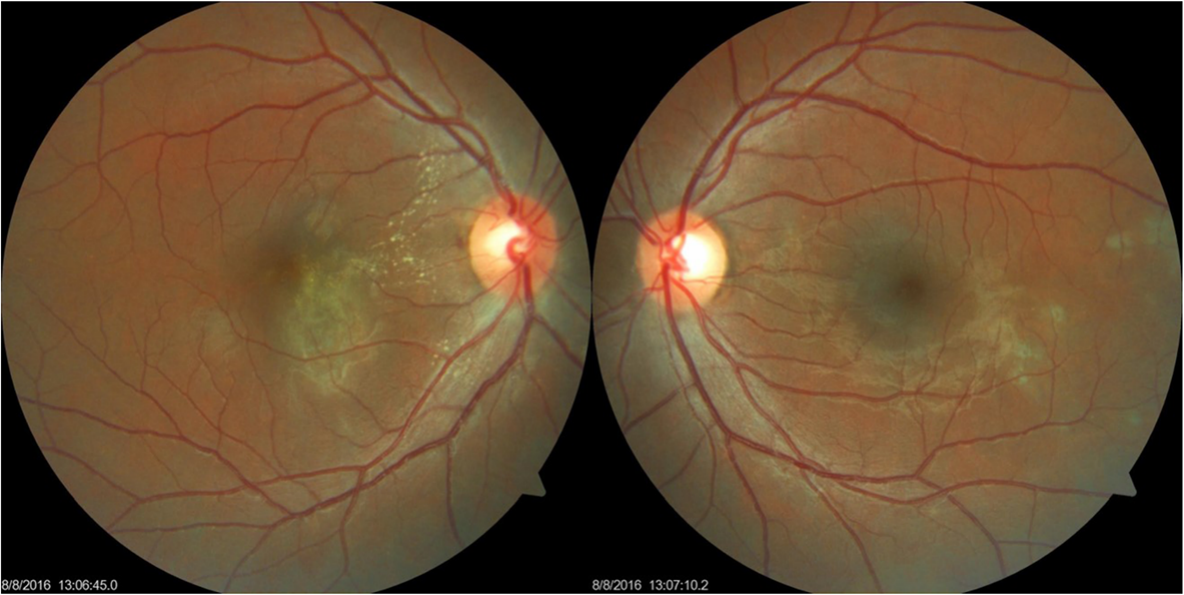
Shown fundus image 2 weeks after presentation. The *right eye* disc hyperaemia appears to have resolved. Presence of hard exudates is as before. The subretinal lesion now appears well defined with marginal reduction in size with pigmentation. The *left eye* disc and macula appear normal. Previously noted lesions now appear as patches of chorioretinal atrophy

A 35-year-old female, already a known case of seropositive rheumatoid arthritis, presented with high-grade fever with chills (temperature as high as 39.4 °C [103 °F]), malaise, and headache for 1 week. She was on leflunomide (20 mg) prescribed by her rheumatologist for last 6 months. She gave a history of acute onset of blurring of vision associated with mild ocular pain in her right eye for 2 days. She was diagnosed positive for NS1 antigen with high dengue serology (IgM). Her serology for chikungunya virus was negative. Her platelet count was at its nadir of 10000/cu.mm. She was transfused with 8 units of platelet and started on other supportive medications. On examination, her best-corrected visual acuity in the right eye was 6/9 and 6/6 in the left eye. Anterior segment examination revealed quiet anterior chamber and anterior vitreous. Fundus examination of the right eye showed yellow deep choroidal lesions at macula, multiple small, hard exudates along papillomacular bundle, and hyperemic disc. There was a small retinal hemorrhage at the temporal margin of disc. The left eye also had hard exudates superonasal and inferotemporal to macula (Fig. 1). Periphery of the fundus in both eyes was normal. There was no relative afferent pupillary defect (RAPD), and her color vision was within normal limits. Fundus fluorescein angiography (FFA) demonstrated these lesions to be early hypofluorescent with late hyperfluorescent staining of the borders. FFA also displayed leakage of the dye from right optic disc and staining of both the discs (Fig. 2). Optical computerized tomography (OCT) revealed disruption and thickening of the inner segment/outer segment (IS/OS) junction with subretinal fluid and intraretinal edema (Fig. 3). She was investigated extensively, and all investigations were non-contributory to the cause of ocular involvement including tuberculin skin test, interferon gamma release assay, serology for syphilis, and high-resolution computed chest tomography (HRCT). She was started on oral prednisolone 60 mg/day after obtaining necessary clearance from physician. She felt subjective improvement in vision after 2 weeks, however, complained of persistence of scotomata, which was more prominent while reading. Fundus examination of her both eyes showed resolved choroiditis (Fig. 4). Her BCVA in both eyes improved to 6/6.

## Discussion

Posterior segment involvement such as retinitis, retinochoroiditis [2], retinal vasculitis [3], and neuroretinitis [4] in dengue has been reported extensively in literature. Retinochoroiditis in dengue was reported by Tabbara [2] who found multiple foci of retinochoroiditis, vasculitis, cotton-wool spots, and retinal hemorrhages in two patients infected with dengue. Pathogenesis of ocular involvement in dengue is not completely understood and believed to be multifactorial. Thrombocytopenia and immune-mediated mechanisms are thought to play an important role [1]. Dengue maculopathy is perhaps the most recognized manifestation of ocular involvement in dengue. Macular edema [3], retinal vasculitis [5], macular hemorrhage, and exudative retinal detachment have been frequently described in association with dengue maculopathy. Foveolitis is characterized by a discrete, well-defined yellow-orange subretinal lesions in the fovea, which usually corresponds to disruption of the outer neurosensory retina and the inner segment/outer segment (IS/OS) junction. Hallmark feature described in these patients was the presence of central scotomata [3, 6], which was reported in absence of macular oedema also. Optic nerve involvement in dengue is not uncommon. In a study of 41 patients of dengue-associated maculopathy by Bacsal et al. [7], 11% of the patients had disc oedema.

The key clinical feature in this case is the presence of choroidal inflammation. The choroiditis in our patient was multifocal, and the patient was investigated to rule out serpiginous-like choroiditis. Her laboratory investigations were non-contributory to the cause of inflammation including tuberculin skin test, interferon gamma release assay and high-resolution computed tomography (HRCT) chest. Presence of hard exudates along papillomacular bundle and OCT findings suggest involvement of overlying retina with subretinal fluid and retinal edema. Unfortunately, we could not perform indocyanine angiography due to systemic condition of the patient. There was no relative afferent pupillary defect, and color vision of the patient was normal. However, deposition of hard exudates in the form of a partial, nasal macular star configuration in the right eye may also be due to optic nerve involvement. The retinal hemorrhage noticed at the temporal margin of the optic disc in right eye can be due to the endothelial cell damage by the dengue virus.

Nevertheless, we believe that our case represents a rare clinical manifestation in wide-spectrum of dengue eye disease. Tabbara [2] reported two patients who developed multiple foci of retinochoroiditis, vasculitis, cotton wool spots, and retinal hemorrhages following dengue fever that resolved spontaneously. Our patient had predominantly choroiditis, and we did not observe any cotton wool spot or vasculitis. One of the patients reported by Tabbara [2], developed macular scarring resulting in poor vision in that eye. Our patient responded well to oral steroid, though she complained of persistence of scotoma even after resolution of her lesions. Exact mechanism of choroiditis in our patient is not clear. Direct cell damage by the dengue virus leading to the apoptosis and dysfunction of the affected cells has been described in literature [8]. Also immune-mediated injury may have played an important role in dengue fever associated choroiditis, similar to foveolitis [6] and dengue-associated maculopathy [7]. Our case highlights the importance of oral steroid in patients with macula threatening lesion in dengue-associated eye disease. In conclusion, our case presents a rare clinical variant of ocular involvement in dengue fever.
